# Searching for Public Health Law’s Sweet Spot: The Regulation of Sugar-Sweetened Beverages

**DOI:** 10.1371/journal.pmed.1001848

**Published:** 2015-07-07

**Authors:** David M. Studdert, Jordan Flanders, Michelle M. Mello

**Affiliations:** 1 Stanford Law School, Stanford, California, United States of America; 2 Center for Health Policy/PCOR, Department of Medicine, Stanford University School of Medicine, Stanford, California, United States of America; 3 Department of Health Research and Policy, Stanford University School of Medicine, Stanford, California, United States of America

## Abstract

David Studdert and colleagues explore how to balance public health, individual freedom, and good government when it comes to sugar-sweetened drinks.

**Summary Points**

- Over the last decade, many national, state, and local governments have introduced laws aimed at curbing consumption of sugar-sweetened beverages (SSBs), especially by children.
- The main regulatory approaches are taxes, restrictions on the availability of SSBs in schools, restrictions on advertising and marketing, labeling requirements, and government procurement and benefits standards.
- Efforts to regulate in this area often encounter stiff opposition, including claims that the laws are inequitable, do not achieve their goals, and have negative economic effects.
- Several lessons can be drawn from the international experience with SSB regulation to date, which may inform future design and implementation of legal interventions to combat noncommunicable disease.

In May 2012, the New York City Board of Health set a limit of 16 ounces on sugary drinks sold in city restaurants, theaters, and food carts [1], triggering international media attention and a firestorm of opposition. A majority of New Yorkers viewed the portion cap as a “bad idea” [2], and three-quarters of Americans opposed it [3]. The soft drink industry embarked on a multimillion-dollar campaign to block the rule [4–6], culminating in a successful legal challenge [7–8].

Sugar-sweetened beverages (SSBs)—including sodas, sweetened teas, and sports, fruit, and energy drinks—have recently become a special target for regulation because of growing evidence regarding their contribution to weight gain and ill health, especially among children [9–12]. New York City’s portion cap is one of hundreds of laws introduced over the last decade by countries, states, and localities around the world to address the problem. The rule’s demise, however, illustrates how public health law interventions can struggle for political and legal traction in the hotly contested space of obesity prevention.

What forms of regulation have been tried? Which ones work? And which are most likely to hit the sweet spot between public health efficacy, political acceptability, and legal survivability? This paper addresses these questions, emphasizing legal and policy developments in the United States, where there has been substantial activity. As soft drink consumption and obesity rise in middle- and lower-income countries, these issues are gaining global salience.

## Regulation of SSBs on the Global Stage

Around the world, governments have initiated SSB regulation in five main areas.

### Taxes

Taxes on SSBs, the most commonly adopted measure, vary widely in type, applicability, and level. SSB taxes may be levied in the form of excise, import, value-added, or sales taxes [13]. While a few countries tax all SSBs and other sugary products, most regimes target certain drinks in ways not tightly tied to caloric content. For example, Brazil recently passed a small excise tax on beer, energy and sports drinks, and fruit juices but spared regular and diet soft drinks. Inconsistencies in the way the United Kingdom’s value-added tax is applied to sweetened beverages have also provoked challenges [14].

A few countries, most notably several South Pacific island nations [15], where obesity rates are among the highest in the world, have introduced very high taxes on SSBs. But most SSB taxes add between 5 and 9 cents per liter. This is well short of the level experts argue is needed to significantly affect consumption and weight outcomes—a sales tax of at least 20% of the container’s price or a specific excise tax of 1 cent per ounce [12,16–18].

At least some of the apparent incoherence of SSB-related taxes stems from the fact that most are designed to raise revenue, not (primarily) to reduce consumption or promote health. Many public health experts see this as a missed opportunity. Evidence from economic modeling studies and a few randomized, controlled trials suggests that properly designed taxes likely would be effective in curbing SSB consumption [19–22].

In the US, there have been many government proposals to introduce or raise taxes—most unsuccessful. The City of Berkeley, California, recently became the first city to pass an SSB tax, a penny-per-ounce excise on soda distributors, but a similar ballot measure in nearby San Francisco failed [23]. At least 22 states have proposed SSB taxes since 2010, but only Washington state passed one at the level recommended by economists, and it was repealed the following year in a voter referendum [24–28]. The beverage industry has invested heavily in public relations firms and “grassroots” organizations to oppose these initiatives [29–30].

### Restrictions on Availability of SSBs in Schools

In at least 30 countries, national or sub-national governments have restricted beverage sales in schools [31]. Some countries (e.g., Cyprus, France, Greece, and Japan) have banned vending machines and permit only water, milk, or 100% fruit juice in schools. Others (e.g., Australia and Costa Rica) have enacted broad bans on SSBs in schools, including sports drinks and sugar-sweetened fruit juices, but permit artificially sweetened drinks.

In the US, most regulation of SSB sales in schools has come from states and local school districts [32]. However, federal influence increased in 2014, with regulations imposing new nutrition standards for food and beverages sold in schools that participate in the National School Lunch Program and School Breakfast Program. The new rules, which apply to all foods sold on school campuses during school hours, prohibit all soft drinks and energy drinks for elementary and middle schools but allow some low-calorie beverages for high school students [33].

Evaluation of school-based restrictions on SSB availability is complicated by the heterogeneous nature of the restrictions and variable enforcement [32]. What evidence exists comes chiefly from US studies, which have found modest or no effect on overall consumption (including consumption outside school) [34–35]. If school-based restrictions help to denormalize SSB consumption, however, they may contribute to lower consumption in the longer term.

### Advertising and Marketing Restrictions

There is broad consensus in the public health community that reducing the influence of advertising is a critical step in addressing the spread of childhood obesity [36–39]. Many countries have taken action in this area, albeit not, in most cases, targeting solely SSBs. France requires all advertising for processed foods and foods or drinks containing added fats, sweeteners, and salt to be accompanied by nutritional messages; violators are taxed 1.5% of their annual expenditure on that advertisement, with tax revenue earmarked for nutritional campaigns [40]. Narrower restrictions, directed at advertising to children, are more common [41–42]. Sweden, Norway, and Quebec, for instance, have enacted complete bans on advertisements of foods, beverages, and toys to children [43]. The UK limits cartoons and incentives in advertisements of foods high in sugar, salt, or fat.

The US and Canada have sought to modulate the behavior of advertisers through a softer approach—mainly via voluntary guidelines and pressure to self-regulate [44–45]. These appear to have had only a modest impact on marketing practices [46]. US regulators face considerable legal barriers in going further [47], including courts’ increasingly expansive interpretations of the scope of protected commercial speech under the First Amendment. Unless judicial currents shift, it will remain extremely difficult to impose restrictions on SSB advertising.

Legal problems have blocked efforts in other countries too. In Brazil, for example, a regulatory initiative designed to restrict advertising of obesogenic foods was derailed by a court challenge asserting that it required a legislative enactment or constitutional amendment, not mere agency action [48]. In Canada, where commercial speech is also constitutionally protected, the Canadian Supreme Court has proven more willing than its American counterpart to defer to legislative judgments that advertising restrictions advance important public health goals [49–50].

### Labeling Rules

The regulation of food and beverage labeling in many countries seeks to boost consumer awareness through a mix of mandatory rules and voluntary codes. The European Union’s recently updated scheme [51], for example, provides for voluntary front-of-pack labeling of energy and nutrients. However, labels that include daily reference intakes for a nutrient other than energy must include the reference intake for sugar, set at 90 g. In the UK, manufacturers are encouraged, not required, to use a "traffic light" system for color-coding percentages of a daily reference intake per 100 g. Products containing sweeteners must include a label indicating this.

Historically, nutritional labeling requirements in the US have been modest: placement is on the back of packaging; information on recommended daily intake (RDI) pertains to fewer nutritional components; and there is no established RDI for sugar, so product labels show volume of sugar content in grams (an unfamiliar metric to many Americans) and without a reference point. However, changes are afoot. The US Food and Drug Administration (FDA) recently proposed rules that would require labels to reflect typical consumption in one sitting—for example, a 20-ounce soft drink bottle would bear the nutritional content for the entire bottle. The new labels would also feature calorie content more prominently [52]. Another noteworthy regulatory development in the US has been mandates—initially local, now nationwide—requiring calorie information on menus and menu boards at chain restaurants and vending machines [53].

Studies to date suggest these changes will have a very modest impact on consumption. Although no evidence is available concerning SSBs specifically, a recent international, systematic review of 17 menu labeling studies concluded that labeling menus with calories only had no effect on calories purchased across the entire population, though it did lead women to select fewer calories [54]. A greater effect was observed when the labels were accompanied by contextual information such as percentage of recommended daily intake, but US law does not require this for restaurant foods.

### Government Procurement and Benefits Standards

Restrictions on which beverages may be purchased using government funds are a less visible form of regulation, but one with potential to change the consumption patterns of large numbers of people. Outside public schools, these standards are most germane in two areas: procurement standards for public institutions (e.g., government agencies, hospitals, and prisons) and restrictions on what recipients of government benefits for the indigent may buy with those funds.

The UK’s Government Buying Standards prohibit central government bodies from procuring SSBs larger than 330 ml and encourage the wider public sector to follow the guidelines [55]. Massachusetts [56–57] and many US counties and cities have adopted nutrition standards for government contracts, but most apply to a limited set of institutions, such as childcare facilities or youth centers.

The question of whether the US government should restrict recipients of Supplemental Nutrition Assistance Program (SNAP) benefits from using them to buy SSBs has provoked heated controversy. SNAP supports food purchases for nearly 50 million low-income Americans. The program’s primary exclusions are hot prepared foods and alcohol. Several states have unsuccessfully petitioned the US government for permission to test SSB exclusions. The latest denial cited logistical feasibility, the lack of a rigorous plan to assess the effects of policy change, and potential for stigmatizing beneficiaries [58].

Restricting the use of SNAP and related benefits to purchase SSBs could help denormalize SSB consumption [59]. On the other hand, some may doubt the effectiveness of SNAP restrictions in light of research indicating that levels of soft drink consumption among youth are insensitive to receipt of SNAP benefits [60].

## Opposition to SSB Regulation

Although the food and beverage industries are the most prominent opponents of the various forms of regulation discussed above, they are regularly joined by interest groups (sometimes industry funded) that champion consumer choice, de-regulation, and smaller government. Opposition to SSB regulation has rallied around five main arguments.

The first and loudest objection tends to be economic: regulation is said to jeopardize the livelihood of companies and workers along the SSB manufacturing and distribution chain [27,61]. This argument can be remarkably effective. For example, Denmark recently decided to scrap its decades-old excise tax on SSBs, citing job and revenue losses and damage to competitiveness from cross-border trade [62].

A second objection points to the inequity of some forms of SSB regulation, especially taxes and restrictions on uses of government benefits. Food and beverage taxes disproportionately burden poorer consumers, who spend a greater share of their household budget on food. Restrictions on SNAP and similar benefits have been criticized for singling out and stigmatizing poor families, potentially discouraging them from participating in government benefits programs [58,63]. Advocacy groups also worry that taxes reduce poor people’s net income without addressing the barriers to accessing healthier food in low-income communities [25]. An extreme version of this argument has been leveled at recently adopted SSB regulations in Mexico, where residents of some poor regions reportedly substitute SSBs for unsafe drinking water [64].

A third critique of SSB regulation is that it will not actually reduce consumption or enhance health. While some research has suggested that raising SSB prices leads consumers to substitute foods that would result in a net increase in sodium and fat consumption [65], other (arguably stronger) studies have found that soft drink taxation does not trigger complete substitution to other sources of fat and is associated with reductions in both caloric intake and obesity [66–67]. Another common refrain is that the regulation is not far-reaching enough to be effective—for example, restrictions on SSBs within schools miss children’s consumption in other settings, and the plaintiffs in New York City’s portion-cap case were quick to point out that consumers could sidestep the rule by purchasing multiple smaller bottles [68].

The ineffectiveness objection is often intertwined with a fourth concern, the arbitrariness of SSB regulation. For instance, a 2011 report on obesity by the Coca-Cola Company cited 45 studies pointing to excess calories from food, rather than SSBs, as the main culprits in causing obesity [69]—the implication being that singling out SSBs for regulation is arbitrary. In the portion-cap litigation, the trial court was troubled by the fact that the rule exempted some convenience stores [7].

The effectiveness and arbitrariness arguments coalesce to create a Catch-22 situation for SSB regulation: the more restrained the restriction is, the greater its vulnerability to criticism for being arbitrary and too weak to be effective, yet sterner laws face objections that they go beyond the evidence and intrude too far into personal liberty.

Fifth, even when regulations do not directly restrict liberty (taxes, for example, merely make some choices more expensive), they are often decried as symptomatic of an encroaching nanny state. Arguments about paternalism have particular traction in the US, where classical liberalism, with its emphasis on autonomy, has long dominated the political sphere. Industrial interests have leveraged this ideological bent to resist regulation, funding putatively pro-consumer organizations to spread the message that attempts to interfere with the choices people make about food and beverages amount to unacceptable paternalism [4].

## Lessons for Global Regulation of SSBs

Several lessons can be gleaned from the American experience with SSB regulation.

First, although policy “nudges” have become fashionable [70], there are dangers in treading too lightly. Strategies such as calorie labels, portion caps, and small beverage taxes preserve consumer freedom but are typically too modest to affect consumer behavior, and such modesty can be recast as arbitrariness. Industry opposition will come whether the intervention is modest or aggressive but should be easier to combat if officials can show their policy is effective. The adage “in for a penny, in for a pound” captures the essence of this insight.

Second, low- and middle-income countries should anticipate that SSB companies will increasingly target them as promising markets for their products [71] and should consider now how to craft a regulatory response. Sales of soft drinks have plateaued in the US, while those in low- and middle-income markets such as Brazil, China, and Mexico are climbing [31,72,73]. China accounted for more than $66 billion in soft drink sales in 2013 [74]. Global experience with tobacco suggests that when the US becomes a more difficult place to sell—due to health concerns, stringent regulation, and price increases necessitated by taxes or litigation—companies push to expand sales overseas. Excise taxes may be a particularly appealing option for resource-poor countries because they offer a double policy win, both curbing soda consumption and generating revenue [12,75,76].

Third, despite strong evidence that restrictions on advertising and marketing have public health benefits, especially for children [47,77], the US has not been able to realize the potential of this avenue of reform. Judicial protection of commercial speech has proved an enduring barrier. Fourth, although evidence of efficacy of many of the legal interventions discussed above is fairly robust, it comes primarily from observational and simulation studies. Stronger evidence to guide regulatory action would be helpful. While large-scale randomized testing of measures such as SSB taxes, restrictions, or labeling approaches is probably impractical, researchers should work with regulators to try to ensure that the rollout of new initiatives is done in ways that make them amenable to rigorous evaluation.

Fifth, savvy regulatory design has tremendous potential. For example, there is growing evidence that taxes that are more salient to consumers, such as those included in a good’s posted price (rather than being levied at the register), are more likely to influence purchasing behavior [78]. “Below the line” regulation also has a place. Government procurement policies and restrictions on use of income support may be especially effective because they remove unhealthy options from the choice set altogether and may also have important denormalizing effects. Public sentiment that government dollars should not be spent on products that harm health and lead to more health care costs down the road may help overcome equity objections to such laws, particularly in countries with very limited health budgets.

Finally, the US experience with obesity-related lawmaking suggests some specific strategies for making the public case for such laws. One is that interventions not clearly presented as measures to protect public health will face stiffer opposition. Earmarking tax revenue for health-related purposes is one way to demonstrate the connection [27,79]. (A recent poll indicated that two-thirds of California voters would support SSB taxes if the resultant revenue went to children’s nutrition and physical activity programs, but only 40% would if it did not [80].) Another strategy is to focus SSB regulations on children. The history of tobacco regulation teaches that child-focused initiatives are more likely to be immediately acceptable and to help increase acceptance of further, later measures. Yet another strategy is procedural: there may be greater tolerance for laws aimed at preventing obesity when policy makers take time to engage the public in the policy-making process [81].

## Conclusion

SSBs are a substantial, preventable contributor to the global burden of obesity and associated health conditions. Yet, the appropriate nature and reach of regulation to curtail SSB consumption remains highly contested. This is a timely reminder of how novel and unsettled public health law remains as a tool for combating noncommunicable disease. Tobacco regulation is well established, at least in developed countries, but stands virtually alone as an example of a multilayered regime trained on a behavioral risk factor for noncommunicable disease. A key question in public health law today is whether analogous regimes will evolve to address other risk factors, and, to the extent they do evolve, what they will look like. The future course of SSB looks set to be a bellwether.

Powerful interest groups are arrayed against efforts to regulate SSBs and other obesogenic products. In its nuanced understanding (and manipulation) of public attitudes, the food and beverage industry has stayed several steps ahead of policy makers. To be successful, regulators must address concerns about effectiveness, fairness, equality, and reasonableness of legal interventions and do so in a way that is articulately and widely communicated. Finding public health law’s sweet spot requires regulatory approaches that are capable both of achieving measurable improvements to public health and of winning victories in courts of law and public opinion.

## Abbreviations

FDA: US Food and Drug Administration
RDI: recommended daily intake
SNAP: Supplemental Nutrition Assistance Program
SSB: sugar-sweetened beverage

## References

[1] New York City Health Code § 81.53.

[2] Grynbaum MM, Connelly M. Sixty percent in City oppose Bloomberg’s soda ban, poll finds. New York Times. 23 August 2012. http://www.nytimes.com/2012/08/23/nyregion/most-new-yorkers-oppose-bloombergs-soda-ban.html?_r=0. Accessed 27 August 2014.

[3] Gollust SE, Barry CL, Niederdeppe J (2014). Americans’ opinions about policies to reduce consumption of sugar-sweetened beverages. Prev Med. 63:52–57. 10.1016/j.ypmed.2014.03.002 24631499

[4] Mello MM, Studdert DM (2014). Making the case for health-enhancing laws after Bloomberg. Hast Ctr Rep. Jan-Feb 44(1). 10.1002/hast.246 24408591

[5] Grynbaum MM. Judge blocks New York City’s limits on big sugary drinks. New York Times, 11 March 2013. http://www.nytimes.com/2013/03/12/nyregion/judge-invalidates-bloombergs-soda-ban.html?hp. Accessed 27 August 2014.

[6] New Yorkers for Beverage Choices. http://nycbeveragechoices.com/. Accessed: 27 August 2014.

[7] New York Statewide Coalition of Hispanic Chambers of Commerce v. New York City Dept. of Health, No. 653684/2012, 2013 WL 1343607 (N.Y. Sup. Ct. March 11, 2013).

[8] New York Statewide Coalition of Hispanic Chambers of Commerce v. New York City Dept. of Health, 23 N.Y.3d 681 (2014).

[9] Malik VS, Pan A, Willett WC, Hu FB (2013). Sugar-sweetened beverages and weight gain in children and adults: a systematic review and meta-analysis. Am J Clin Nutr. 98:1084–102. 10.3945/ajcn.113.058362 23966427 PMC3778861

[10] Malik VS, Schulze MB, Hu FB (2006). Intake of sugar-sweetened beverages and weight gain: a systematic review. Am J Clin Nutr. 84:274–88. 16895873 10.1093/ajcn/84.1.274 PMC3210834

[11] Vartanian LR, Schwartz MB, Brownell KD (2007). Effects of soft drink consumption on nutrition and health: a systematic review and meta-analysis. Am J Pub Health. 97:667–75. 17329656 10.2105/AJPH.2005.083782 PMC1829363

[12] Brownell KD, Farley T, Willett WC, Popkin BM, Chaloupka FJ, et al (2009). The public health and economic benefits of taxing sugar-sweetened beverages. N Engl J Med. 361:1599–1605. 10.1056/NEJMhpr0905723 19759377 PMC3140416

[13] Chriqui FJ, Chaloupka FJ, Powell LM, Edison SS (2013). A typology of beverage taxation: Multiple approaches for obesity prevention and obesity prevention-related revenue generation. J Pub Health Pol. 34:403–423. 10.1057/jphp.2013.17 PMC3730238 23698157

[14] Pidd H, Hawke. Innocent loses fight over VAT charged on smoothies—but not raw fruit. The Guardian. 22 November 2010. http://www.theguardian.com/politics/2010/nov/22/innocent-smoothies-challenge-vat-ruling. Accessed December 4, 2014.

[15] Thow AM, Quested C, Juventin L, Kun R, Khan AN, et al (2010). Taxing soft drinks in the Pacific: implementation lessons for improving health. Health Promot Int’l. 26(1):55–64. 10.1093/heapro/daq057 20739326

[16] Mytton O, Rayner M. Health Related Food Taxes and Subsidies, BHF Health Promotion Research Group, University of Oxford. http://www.aomrc.org.uk/doc_view/9578-british-heart-foundation-health-promotion-research-group-dept-of-public-health-oxford. Accessed 27 August 2014.

[17] Briggs A (2013). Overall and income specific effect on prevalence of overweight and obesity of 20% sugar-sweetened drink tax in UK: econometric and comparative risk assessment modeling study. BMJ. 347: f6189 10.1136/bmj.f6189 24179043 PMC3814405

[18] Powell LM, Chriqui JF, Khan T, Wada R, and Chaloupka FJ (2013). Assessing the potential effectiveness of food and beverage taxes and subsidies for improving public health: a systematic review of prices, demand and body weight outcomes. Obesity Rev. 14:110–128. 10.1111/obr.12002 PMC3556391 23174017

[19] Mytton OT, Clarke D, Rayner M (2012). Taxing unhealthy food and drinks to improve health. BMJ. 344:e2931 10.1136/bmj.e2931 22589522

[20] Chaloupka FJ, Powell LM, Chriqui JF (2011). Sugar-sweetened beverages and obesity: the potential impact of public policies. J Pol’y Anal Mgmt. 30:644–665. 10.1002/pam.20587 21774165

[21] Buhler S, Raine KD, Arango M, Pellerin S, Neary NE (2013). Building a strategy for obesity prevention one piece at a time: the case of sugar-sweetened beverage taxation. Can J Diabet. 37(2):97–102. 10.1016/j.jcjd.2013.03.025 24070799

[22] Waterlander WE, Mhurchu CN, Steenhuis IHM (2014). Effects of a price increase on purchases of sugar sweetened beverages. Results from a randomized controlled trial. Appetite. 78:32–39. 10.1016/j.appet.2014.03.012 24667153

[23] Knight H. Why Berkeley passed a soda tax and S.F. didn’t. San Francisco Chronicle. November 7, 2014. http://www.sfgate.com/bayarea/article/Why-Berkeley-passed-a-soda-tax-and-S-F-didn-t-5879757.php.

[24] Washington Post Editors. Council all but kills soda tax. Washington Post. 20 May 2010. http://voices.washingtonpost.com/dc/2010/05/council_all_but_kills_soda_tax.html. Accessed 27 August 2014.

[25] Hartocollis A. Failure of state soda tax plan reflects power of an antitax message. New York Times, July 2, 2010. http://www.nytimes.com/2010/07/03/nyregion/03sodatax.html?_r=0. Accessed 27 August 2014.

[26] Kamerow D (2010). The case of the sugar sweetened beverage tax. BMJ. 341:c3719 10.1136/bmj.c3719 20630952

[27] Jou J, Niederdeppe J, Barry CL, Gollust SE (2014). Strategic messaging to promote taxation of sugar-sweetened beverages: lessons from recent political campaigns. Am J Pub Health. 104:847–53. 24625177 10.2105/AJPH.2013.301679 PMC3987612

[28] Rudd Center for Food Policy and Obesity. Sugar-sweetened beverage initiatives since 2009. Jan. 2013. http://yaleruddcenter.org/resources/upload/docs/what/policy/SSBtaxes/SSB_Initiatives_since_2009.pdf Accessed 27 August 2014.

[29] Steinmetz K. Big soda fights Bay Area tax proposals. Time. Nov 3, 2014. http://time.com/3552008/soda-tax-san-francisco-berkeley/ Accessed 12 May 2015.

[30] Bauerlein V, McKay B. Soda tax uncaps a fight. Wall Street Journal. 5 23, 2010 http://www.wsj.com/articles/SB10001424052748704904604575262530291194198 Accessed 12 May 2015.

[31] Hawkes C (2010). The worldwide battle against soft drinks in schools. Am J Prev Med. 38:457–61. 10.1016/j.amepre.2010.01.011 20307815

[32] Mello MM, Pomeranz J, Moran P (2008). The interplay of public health law and industry self-regulation: the case of sugar-sweetened beverage sales in schools. Am J Pub Health. 98(4):595–604. 17901427 10.2105/AJPH.2006.107680 PMC2376983

[33] National School Lunch Program and School Breakfast Program—Nutrition Standards for All Foods Sold in Schools as Required by the Healthy, Hunger-Free Kids Act of 2010; Interim final rule. Fed Regist. 6 28, 2013;78(125): 39068–39120. 23833807

[34] Taber DR, Chriqui JF, Powell LM, Chaloupka FJ (2012). Banning all sugar-sweetened beverages in middle schools: reduction of in-school access and purchasing but not overall consumption. Arch Pediatr Adolesc Med. 166(3):256–262. 10.1001/archpediatrics.2011.200 22064875 PMC4077154

[35] Fletcher JM, Friswold D, Tefft N (2010). Taxing soft drinks and restricting access to vending machines to curb child obesity. Health Aff. 29:1059–66. 10.1377/hlthaff.2009.0725 20360172

[36] Institute of Medicine (2013). Challenges and opportunities for change in food marketing to children and youth: workshop summary. Washington, DC: National Academies Press. 24872975

[37] World Health Organization (2004). Global strategy on diet, physical activity and health. 2004. http://www.who.int/dietphysicalactivity/strategy/eb11344/strategy_english_web.pdf?ua=1. Accessed 27 August 2014.

[38] World Health Organization (2006). Marketing of food and non-alcoholic beverages to children: report of a WHO forum and technical meeting. Geneva: World Health Organization, 2006. http://whqlibdoc.who.int/publications/2006/9241594918_eng.pdf?ua=1. Accessed 27 August 2014.

[39] Institute of Medicine (2006). Food marketing to children and youth: threat or opportunity? Washington, DC: National Academies Press

[40] Nutritional health warnings: just for show. Prescrire Int 2007;16 (92):261 http://english.prescrire.org/en/80/160/46239/0/PositionDetails.aspx. Accessed 27 August 2014.

[41] Hawkes C (2007). Marketing food to children: changes in the global regulatory environment 2004–2006. Geneva: World Health Organization, 2007. http://www.who.int/dietphysicalactivity/regulatory_environment_CHawkes07.pdf. Accessed 17 August 2014.

[42] Hawkes C. Marketing food to children: the global regulatory environment. Geneva: World Health Organization, 2004 http://whqlibdoc.who.int/publications/2004/9241591579.pdf. Accessed 14 August 2014.

[43] Oommen VG, Anderson PJ (2008). Policies on restriction of food advertising during children’s television viewing times: an international perspective. http://eprints.qut.edu.au/13646/1/Policies_on_Food_Advertising.pdf. Accessed 27 August 2014.

[44] White House Task Force on Childhood Obesity (2010). Solving the problem of childhood obesity within a generation: White House Task Force on Childhood Obesity report to the President. May 2010. http://www.letsmove.gov/sites/letsmove.gov/files/TaskForce_on_Childhood_Obesity_May2010_FullReport.pdf. Accessed 27 August 2014. 10.1089/bfm.2010.9980 20942695

[45] Interagency Working Group on Food Marketing to Children (2011). Preliminary proposed nutrition principles to guide industry self-regulatory efforts. https://www.cspinet.org/new/pdf/IWG_food_marketing_proposed_guidelines_4.11.pdf Accessed 27 August 2014.

[46] Dietz WH (2013). New strategies to improve food marketing to children. Health Aff. 32:1652–58. 10.1377/hlthaff.2012.1294 24019372

[47] Mello MM (2010). Federal Trade Commission regulation of food advertising to children: possibilities for a reinvigorated role. J Health Polit Pol’y Law. 35(2):227–76. 10.1215/03616878-2009-051 20388868

[48] Jaime PC, da Silva CF, Gentil PC, Claro RM, Monteiro CA (2013). Brazilian obesity prevention and control initiatives. Obes Rev. 14(Suppl. 2): 88–95. 10.1111/obr.12101 24102701

[49] Berman ML (2013). Commercial speech law and tobacco marketing: a comparative discussion of the United States and Canada. Am J Law Med. 39(2–3):218–36. 23815029 10.1177/009885881303900202

[50] Irwin Toy Ltd. v. Attorney General of Quebec, [1989] 1 SCR 927.

[51] European Union Regulation No. 1169/2011. http://eur-lex.europa.eu/legal-content/EN/TXT/?qid=1418204194755&uri=CELEX%3A02011R1169-20140219. Accessed 13 May 2015.

[52] US Food and Drug Administration (2014). 21 CFR Part 101 (May 27 2014). http://www.regulations.gov/#!documentDetail;D=FDA-2004-N-0258-0006 Accessed 27 August 2014/

[53] Patient Protection and Affordable Care Act, P.L. 111–148, sec. 4205 (2010).

[54] Sinclair SE, Cooper M, Mansfield ED (2014). The influence of menu labeling on calories selected or consumed: a systematic review and meta-analysis. J Acad Nutr Dietetics. 114(9):1375–88. 10.1016/j.jand.2014.05.014 25037558

[55] UK Department of Environment, Food and Rural Affairs (2011). Guidance on government buying standards for food and catering services. http://sd.defra.gov.uk/documents/GBS-guidance-food.pdf. Accessed 27 August 2014.

[56] Gov. Deval L. Patrick, Exec. Ord. 509 (MA 2009). http://www.mass.gov/courts/docs/lawlib/eo500-599/eo509.pdf Accessed 27 August 2014.

[57] Mass in Motion. Massachusetts State Agency Food Standards. http://www.mass.gov/eohhs/docs/dph/com-health/nutrition-phys-activity/eo509-state-agency-food-standards.pdf. Accessed 27 August 2014.

[58] Brownell KD, Ludwig DS (2011). The Supplemental Nutrition Assistance Program, soda, and USDA policy: who benefits? JAMA. 306:1370–71. 10.1001/jama.2011.1382 21954481

[59] Barnhill A (2011). Impact and ethics of excluding sweetened beverages from the SNAP program. Am J Pub Health. 101:2037–43. 21566025 10.2105/AJPH.2011.300225 PMC3222381

[60] Fernandes MM (2012). Effect of the Supplemental Nutrition Assistance Program (SNAP) on frequency of beverage consumption among youth in the United States. J Acad Nutr Diet. 112:1241–46. 10.1016/j.jand.2012.03.033 22682882

[61] Niederdeppe J, Gollust SE, Jarlenski MP, Nathanson AM, Barry CL (2013). News coverage of sugar-sweetened beverage taxes: pro- and antitax arguments in public discourse. Am J Pub Health. 103(6):e92–e98. 23597354 10.2105/AJPH.2012.301023 PMC3698716

[62] Health Be Damned: Denmark hopes cheaper soda will boost economy. Spiegel International, Apr. 23, 2013. http://www.spiegel.de/international/europe/denmark-to-repeal-tax-on-soda-and-beer-to-limit-cross-border-shopping-a-895857.html. Accessed 27 August 2014.

[63] Barnhill A, King KF (2013). Evaluating equity critiques in food policy: the case of sugar-sweetened beverages. J Law Med Ethics. 41(1):301–9. 10.1111/jlme.12020 23581672

[64] Stier J (2013). With its soda tax, Mexico repeats the mistakes of Mayor Bloomberg. Forbes, 10 October 2013. http://www.forbes.com/sites/realspin/2013/10/10/with-its-soda-tax-mexico-repeats-the-mistakes-of-mayor-bloomberg/. Accessed 27 August 2014.

[65] Zhen C, Finkelstein EA, Nonnemaker J, Karns S, Todd JE (2013). Predicting the effects of sugar-sweetened beverage taxes on food and beverage demand in a large demand system. Am J Agric Econ. 96(1):1–25. 10.1093/ajae/aat049 PMC4022288 24839299

[66] Finkelstein EA, Zhen C, Bilger M, Nonnemaker J, Farooqui AM, et al (2013). Implications of a sugar-sweetened beverage (SSB) tax when substitutions to non-beverage items are considered. J Health Econ. 32:219–39. 10.1016/j.jhealeco.2012.10.005 23202266

[67] Cabrera Escobar MA, Veerman JL, Tollman SM, Bertram MY, Hofman KJ (2013). Evidence that a tax on sugar sweetened beverages reduces the obesity rate: a meta-analysis. BMC Pub Health. 13:1072. 24225016 10.1186/1471-2458-13-1072 PMC3840583

[68] Wilson BM, Stolarz-Fantino S, Fantino E (2013). Regulating the way to obesity: unintended consequences of limiting sugary drink sizes. PLoS ONE. 8(4):e61081 10.1371/journal.pone.0061081 23593397 PMC3622664

[69] The Coca-Cola Company (2011). Our position on obesity—including supporting science and facts, Sept. 2011. http://assets.coca-colacompany.com/c2/a7/2f6eab904c7cbf3fcbc0646bd988/Our%20Position%20on%20Obesity.pdf. Accessed: 27 August 2014.

[70] Thaler RH, Sunstein CR (2008). Nudge: improving decisions about health, wealth, and happiness. New Haven: Yale University Press.

[71] Stuckler D, McKee M, Ebrahim S, Basu S (2012). Manufacturing epidemics: the role of global producers in increased consumption of unhealthy commodities including processed foods, alcohol, and tobacco. PLoS Med. 9(6):e1001235 10.1371/journal.pmed.1001235 22745605 PMC3383750

[72] Basu S, McKee M, Galea G, Stuckler D (2013). Relationship of soft drink consumption to global overweight, obesity, and diabetes: a cross-national analysis of 75 countries. Am J Pub Health. 103:2071–77. 23488503 10.2105/AJPH.2012.300974 PMC3828681

[73] Monteiro CA, Cannon G (2012). The impact of transnational “Big Food” companies on the South: a view from Brazil. PLoS Med. 9(7):e1001252 10.1371/journal.pmed.1001252 22802732 PMC3389019

[74] Euromonitor International. Global soft drink market grows 2.1% despite rising health concerns, 5 February 2014. http://www.reuters.com/article/2014/02/05/idUSnMKWMx6N2a+1e4+MKW20140205 Accessed 27 August 2014.

[75] Mello MM, Cohen IG (2012). The taxing power and the public’s health. N Engl J Med. 367:1777–9. 10.1056/NEJMp1209648 23075142

[76] Basu S, Vellakkal S, Agrawal S, Stuckler D, Popkin B, et al (2014). Averting obesity and type 2 diabetes in India through sugar-sweetened beverage taxation: an economic-epidemiologic modeling study. PLoS Med. 11(1):e1001582 10.1371/journal.pmed.1001582 24409102 PMC3883641

[77] Bogart WA (2013). Law as a tool in “the war on obesity”: useful interventions, maybe, but first, what’s the problem? J Law Med & Ethics. 41(1):28–41. 10.1111/jlme.12003 23581655

[78] Goldin J (2012). Sales tax not included: designing commodity taxes for inattentive consumers. Yale Law J. 122(1):258–301.

[79] Pomeranz JL (2012). Advanced policy options to regulate sugar-sweetened beverages to support public health. J Pub Health Pol’y. 33(1):75–88. 10.1057/jphp.2011.46 21866177

[80] Field Research Corporation. The Field Poll, Release #2436, 14 February 2013. http://field.com/fieldpollonline/subscribers/Rls2436.pdf Accessed 27 August 2014.

[81] Morain S, Mello MM (2013). Survey finds public support for legal interventions directed at health behavior to fight noncommunicable disease. Health Aff. 32:486–496. 10.1377/hlthaff.2012.0609 23459727

